# Type 2 Diabetes Modulates Mesenchymal Stem Cell Response to Advanced Glycation End Products and N-Acetylcysteine Antioxidant Effect

**DOI:** 10.3390/pharmaceutics18050595

**Published:** 2026-05-13

**Authors:** Rebecca Landon, Ji Ding, William Ndjidda Bakari, Nathanael Larochette, Hanane El-Hafci, Olivier Thibaudeau, Abolfazl Barzegari, Virginie Gueguen, Graciela Pavon-Djavid, Fani Anagnostou

**Affiliations:** 1 Université Paris Cité, CNRS UMR7052, INSERM U1271, ENVA, B3OA, F-75010 Paris, France; ji.ding@cnrs.fr (J.D.); william.bakari@cnrs.fr (W.N.B.); nathanael.larochette@cnrs.fr (N.L.); hanane.el-halfci@univ-paris-diderot.fr (H.E.-H.); 2 Laboratory for Vascular Translational Science (LVTS), Cardiovascular Bioengineering, Université Sorbonne Paris Nord, Inserm U1148, F-93430 Villetaneuse, France; barzegari.abolfazl@gmail.com (A.B.); virginie.gueguen@univ-paris13.fr (V.G.); graciela.pavon@univ-paris13.fr (G.P.-D.); 3 Hôpital Bichat-Claude-Bernard, Plateau de Morphologie UMR 1152, Université Paris Cité, F-75018 Paris, France; olivier.thibaudeau@inserm.fr; 4 Department of Medical Biotechnology, Faculty of Advanced Medical Sciences, Tabriz University of Medical Sciences, Tabriz 51656-65931, Iran; 5 Department of Odontology, Assistance Publique Hôpitaux de Paris (AP-HP), Pitié-Salpêtrière Hospital, F-75013 Paris, France

**Keywords:** diabetes, bone marrow, bone marrow mesenchymal stem cells (BMMSCs), mesenchymal stem cell, advanced glycated end-products (AGEs), N-acetylcysteine (NAC), antioxidant

## Abstract

**Background**: Advanced glycation end products (AGEs) and oxidative stress (OS) have been linked to bone complications related to type 2 diabetes mellitus (T2DM). However, the effects of AGEs and OS on bone marrow mesenchymal stromal cells (BMMSCs), which play a key role in bone homeostasis and repair, remain unclear. **Objectives**: This study aimed to investigate the effects of AGEs on BMMSCs function and the ability of N-acetylcysteine (NAC) to alleviate AGE-induced OS in a T2DM context. **Methods**: Bone marrow (BM) and BMMSCs were isolated from Zucker diabetic fatty (ZDF) rats, which serve as a T2DM model, and their lean littermates (ZL, controls) at 24 weeks of age. **Results**: The results show that long-standing T2DM leads to changes in the BM’s cellular composition and BMMSCs function that are distinct from age-related changes. In vitro, AGEs decreased BMMSCs viability, proliferation, and migration. The effects of AGEs were stronger in BMMSCs derived from a T2DM microenvironment. In both T2DM- and ZL-BMMSCs, AGEs induced cytoplasmic ROS, which was differentially reduced by NAC. The effect of NAC on T2DM-BMMSCs was greater when the cells were pre-treated with NAC 24 h before exposure to AGEs, whereas simultaneous exposure to both resulted in a smaller effect. **Conclusions**: These results show that AGEs impair BMMSCs expansion and functionality. AGE-induced ROS generation may be a critical factor in this impairment, while NAC was able to reduce OS in BMMSCs from a T2DM context. These findings highlight the vicious negative effects of the T2DM microenvironment on BMMSCs and underscore the need for further studies to better understand the underlying mechanisms and to explore strategies aimed at mitigating OS in the T2DM context.

## 1. Introduction

Type 2 diabetes mellitus (T2DM) is a significant global health issue due to its increasing incidence and the widespread prevalence of its complications [1]. T2DM is a chronic metabolic disease leading to critical complications, including retinopathy, peripheral neuropathy, nephropathy, cardiovascular disease, and non-healing chronic wounds [2]. Bone is another target tissue of T2DM. It increases the risk of osteoporosis and fractures and impairs bone healing [3].

T2DM affects bone tissue at the structural, cellular, and molecular levels [4]. Specifically, besides vascular complications, T2DM induces alterations in the bone marrow, affecting both the vascular and endosteal niches as well as bone cells, including bone marrow mesenchymal stromal cells (BMMSCs) [5,6]. BMMSCs have the capacity to proliferate and differentiate into a multilineage [6]. They home in on sites of injury and play a key role in bone homeostasis and repair [7,8,9,10]. Moreover, thanks to their low immunogenicity and secretome, they have been highlighted as promising candidates in regenerative medicine. However, several studies have reported that in the T2DM microenvironment, several functions of BMMSCs are altered [6]. The T2DM microenvironment is complex, and the contributions of various factors (hyperglycemia, inflammation, hypoxic conditions, advanced glycation end products (AGEs), oxidative stress (OS)) underlying BMMSCs impairment remain under investigation. Specifically, chronic hyperglycemia promotes the accumulation of AGEs, which are highly involved in the pathogenesis of many T2DM complications [11]. AGEs accumulate as a normal process of aging and, as a result of hyperglycemia, have been shown to induce changes in matrix components [12,13,14], including those in bone tissue. However, the effects on BMMSCs in the T2DM context are not yet clear, and the underlying mechanisms remain elusive.

AGEs’ pathological effects on various cell types are related to their ability to promote OS [15,16,17], and several studies have reported that in T2DM, the redox balance of cells shifts towards a more pro-oxidant state [18]. AGEs, in addition to direct changes in protein structure and function, cause damage by binding to receptors for advanced glycation products (RAGEs) and mediating the intracellular production of reactive oxygen species (ROS) [15,16]. ROS play an essential role in cellular homeostasis and regulate a range of MSC signaling pathways [19]. Increased intracellular ROS (iROS) is associated with BMMSCs dysfunction [20]. In contrast, ROS scavengers such as mito-TEMPO or N-Acetylcysteine (NAC) have been reported to alleviate OS-induced damage in BMMSCs harvested from non-diabetic environments [21,22]. Moreover, NAC has been shown to have beneficial effects on non-diabetic BMMSCs, but its effects on AGE-induced OS in the context of T2DM remain poorly investigated.

Thus, the present study aimed to investigate the effects of AGEs on select functions and ROS production in BMMSCs harvested from both long-standing T2DM and non-diabetic microenvironments, as well as the antioxidant potential of NAC on AGE-induced ROS. In this study, we used ZDF rats and their lean littermates at 24 weeks of age. This T2DM model exhibits most of the metabolic, microvascular and musculoskeletal features of T2DM reported in humans, including impaired vascular and bone repair [23,24,25] as well as altered BMMSCs functionality [20,26]. Unraveling AGE-induced OS helps improve understanding of the impact of the T2DM microenvironment on BMMSCs and provides new insights into protecting these cells from OS in the T2DM context.

## 2. Materials and Methods

### 2.1. Animal Model

Adult 24-week-old male obese Zucker fa/fa (ZDF) rats and age-/sex-matched non-diabetic lean fa/+ littermates (ZL) were purchased from Charles River (L’Arbresle, France). Animals were housed under standard conditions, manipulated, and euthanized according to the European Community Standards on Laboratory Animal Care. The animal protocols were approved by the Paris-Diderot University Ethics Committee (intern referenceFA-1-2020). On sacrifice day, blood was collected, and humeri, tibiae, and femurs were dissected. Diabetic status was assessed using glycated hemoglobin in blood and plasma glucose/fructosamine (Architect Automat; Abbott, Rungis, France). Plasma alkaline phosphatase, cholesterol, triglycerides, ultra-HDL, LDL-C, calcium, potassium, phosphorus, and sodium were measured using standard methods (Architect Automat; Abbott, France).

### 2.2. Histology and Immunohistochemistry

To assess bone marrow histology, the humeri from ZDF and ZL rats were fixed in 10% neutral-buffered formalin for 24 h, decalcified with EDTA (177 g/L, pH 7.0–7.3; Osteosoft, Merck KGaA, Darmstadt, Germany) at 4 °C for 4 weeks and paraffin-embedded. For adiposity analysis, sagittal sections (5 µm) were stained with Masson’s trichrome. Images from two sections per sample were blindly analyzed at ×40 magnification using Fiji (ImageJ, version 1.53) in two regions of interest (1310.68 × 822.81 µm) at 1 mm below the growth plate to determine adipocyte number, diameter, and surface area. For osteocyte analysis, 5 µm mid-diaphysis cross-sections underwent cleaved caspase-3 immunohistochemistry following the established lab protocol: citrate buffer treatment, TBST blocking (1 h, 37 °C), and overnight incubation at 4 °C with primary antibody (Cleaved Caspase-3 [Asp175], #9661, Cell Signaling Technology, Danvers, MA, USA; 1:200 in SignalStain^®^ Antibody Diluent #8112), followed by SignalStain^®^ Boost IHC Detection Reagents (HRP, Rabbit #8114S) and the DAB Substrate Kit (#8058S). The sections were counterstained with Harris hematoxylin and mounted. Two sections per sample were blindly analyzed at ×40 magnification across six regions (171.23 × 171.23 µm) on endosteal/periosteal surfaces using Fiji (ImageJ) to quantify total osteocytes, empty lacunae and cleaved caspase-3-positive osteocytes per bone area; caspase-3-positive lacunae were normalized to total lacunae per ROI.

### 2.3. Isolation of Rat Bone Marrow Mesenchymal Stem Cells

BMMSCs were isolated from the femurs and tibiae as described previously [20]. Bones were harvested, epiphyses were removed, and bone marrow was flushed with alpha-Modified Eagle’s Medium (αMEM) (Invitrogen, Cergy Pontoise, France). Cells from ZL- and ZDF rats were seeded separately at 5 × 10^5^ cells/cm^2^ in αMEM supplemented with 10% (*v*/*v*) fetal calf serum (FCS) and 1% (*v*/*v*) penicillin/streptomycin (ATB) (PAA Laboratories GmbH, Pasching, Austria) (complete medium) and cultured at 37 °C in 5% CO_2_. Non-adherent cells were removed after 2 days; fibroblastic colonies (CFU-F) were formed on day 5 and pooled on day 12 (passage 1). Cells were expanded to 70% confluence, trypsinized with 0.005% trypsin/EDTA, and seeded at 1 × 10^4^ cells/cm^2^; the medium was changed twice weekly. BMMSCs (passages 3–4) were used for all the experiments. The BMMSCs’ phenotypes were confirmed via flow cytometry for CD29 (clone Hmb1–1; eBioscience, San Diego, CA, USA) and CD90 markers (clone HIS51; Life Technologies, Carlsbad, CA, USA), as well as the absence of CD45 (clone OX1; eBioscience, San Diego, CA, USA), and via osteogenic and adipogenic differentiation. As previously described, ZDF-derived cells (passage 2) showed a similar MSC marker profile (including CD90 and CD105) to ZL cells, with no CD45 expression [20].

### 2.4. Advanced Glycation End Products’ Effects on BMMSCs Harvested from ZL and ZDF Rats

#### 2.4.1. Cytotoxicity

BMMSCs from ZL and ZDF rats were separately seeded at 1 × 10^4^ cells/cm^2^ on 96-well plates and cultured in αMEM + 2% FCS + 1% ATB for 24 h. The medium was then replaced by serum-free αMEM containing 0–200 µg/mL advanced glycation end products–bovine serum albumin (AGEs-BSA) (CALBIOCHEM, Merck KGaA, Darmstadt, Germany) for 48 h. After medium removal, cells were rinsed with PBS, dried, and frozen at −70 °C. Cell number was quantified using the CyQUANT^®^ assay (Molecular Probes, Invitrogen™, Thermo Fisher Scientific, Waltham, MA, USA) according to the manufacturer’s instructions.

#### 2.4.2. Cell Survival and Morphology

ZDF- and ZL-BMMSCs were seeded at 1 × 10^4^ cells/cm^2^ in 8-well chamber slides (Lab-Tek^®^ II, Nunc™, Thermo Fisher Scientific, Waltham, MA, USA) in αMEM + 2% FCS. After 24 h, the medium was replaced with serum-free αMEM containing 0–200 µg/mL AGEs-BSA for 48 h. Adherent cells were fixed with 4% formaldehyde, permeabilized with 0.1% Triton X-100 in PBS, stained with Phalloidin (1/100 in PBS) (Alexa Fluor™ Plus 555 Phalloidin, Thermo Fisher), and mounted with coverslips. Four random fields per condition were acquired by confocal microscopy, and adherent cells were counted using ImageJ (NIH, Bethesda, MD, USA).

#### 2.4.3. Cell Proliferation

BMMSCs were seeded at 1 × 10^4^ cells/cm^2^ in 96-well plates in αMEM + 2% FCS + 1% ATB. After adhesion, the medium was changed to complete αMEM + 10% FCS + 1% ATB containing 100–200 µg/mL AGEs-BSA, and proliferation was monitored for up to 10 days. At each time point, cells were rinsed with PBS, plates were stored at −70 °C, and the cell number was assessed with the CyQUANT^®^ assay as described above.

#### 2.4.4. Cell Migration

BMMSCs migration was assessed using 24-well Boyden chambers (Corning Costar, Cambridge, MA, USA). ZDF or ZL cells (75 × 10^3^ cells/100 µL) in serum-free αMEM were placed in the upper compartment, and 0.6 mL of complete medium containing 100 µg/mL AGEs-BSA was added to the lower compartment. Cells were incubated overnight at 37 °C, 5% CO_2_. Cells that had migrated to the underside of the membrane were fixed with 4% paraformaldehyde and stained with May-Grunwald Giemsa. The membranes were excised, mounted on glass slides, and imaged (VHX-2000; Keyence, Osaka, Japan). Cells were counted in five randomly selected fields per membrane using ImageJ.

#### 2.4.5. RT-PCR

BMMSCs were cultured in a complete medium supplemented with increasing concentrations of AGEs (0–100 μg/mL). Total RNA was extracted using TRIzol (Life Technologies) according to the manufacturer’s protocols. Real-time PCR was performed as previously described [20] with the following Taqman primers: Recombinant Interleukin 1 beta (IL1beta, Rn 00580432_m1), TNF superfamily member 11 (RANKL, Rn 00589289_m1), chemokine C-X-C motif ligand 1 (Cxcl1, Rn 00578225_m1), Runt-related transcription factor 2 (Runx2, Rn 01512298_m1), alkaline phosphatase (PDZ and LIM domain 3 ALP, Rn 01516028_m1), peroxisome proliferator-activated receptor gamma (Pparg, Rn 00440940_m1), insulin-like growth factor 1 (IGF-1, Rn 00710306_m1), hypoxia-inducible factor 1 (Hif-1a Rn 00577560_m1), and advanced glycation end product receptor (Ager Rn 00584249_m1), with 18S (Hs 99999901_s1) RNA as the endogenous control.

#### 2.4.6. Reactive Oxygen Species (ROS) Measurement

ROS were quantified using oxidation-sensitive probes: the dichloro-dihydro-fluorescein diacetate DCFH-DA (DCFH-DA, Thermo Fisher) was used for intracellular ROS (iROS) and the dihydroethidium probe (DHE Thermo Fisher) was used for mitochondrial ROS (mROS) detection. Cells were seeded in complete culture medium on 96-well plates (1 × 10^4^ cells/well), cultured for 24 h, rinsed with PBS, exposed to AGEs-BSA (100 µM, 15 min) and incubated in the dark with DCFH-DA (5 µM) or DHE (5 µM) at 37 °C for 30 min. mROS were induced by antimycin A (20 μM). Fluorescence DCFHDA (485/530 nm) and DHE (500/580 nm) were read after 60 min in a plate reader fluorimeter (FL600).

To test N-acetylcysteine (NAC)’s protection against AGE-induced ROS production, cells were either pre-treated overnight with NAC (2.5–10 mM) before the OS induction or co-treated with 10 mM NAC during OS induction. After supernatant removal, cells were incubated with DCFH-DA (10 µM, 1 h, 37 °C, phenol red-free αMEM). OS was also induced by the addition of the stressors AAPH (25 mM) and t-BuOOH (100 μM), as previously published [27]. DCFH-DA fluorescence (485/530 nm), recorded every 5 min for 60 min, was taken as a direct readout of ROS production by ZDF- and ZL-BMMSCs.

### 2.5. Statistical Analyses

Experiments were performed in triplicate across at least three independent cell preparations, with data expressed as mean ± SEM. Time-dependent data were analyzed with ANOVA for unpaired two-tailed samples, while time-independent data were analyzed using Student’s *t*-tests, comparing treatments to respective controls. Differences were considered significant at *p* < 0.05.

## 3. Results

### 3.1. Biochemical Parameters and Body Weight in Long-Standing Diabetic ZDF Rats

Compared to ZL rats, ZDF rats showed a significant (*p* < 0.05) increase in serum glucose, fructosamine, and HbA1c levels (by 1.46-fold, 0.46-fold, and 3.10-fold, respectively) (Table 1). Besides dysglycemia, they also showed significantly higher levels of dyslipidemia markers (*p* < 0.05), including total cholesterol (1.46-fold), triglycerides (1.46-fold), LDL-C (1.33-fold), and ultra-HDL (1.75-fold). In ZDF rats, the circulating alkaline phosphatase level was significantly higher (by 3.40-fold) than that of ZL rats, whereas calcium, phosphorus, sodium, and potassium levels were similar.

### 3.2. Long-Standing T2DM Affects the Microenvironment of BMMSCs

**Figure 1:**
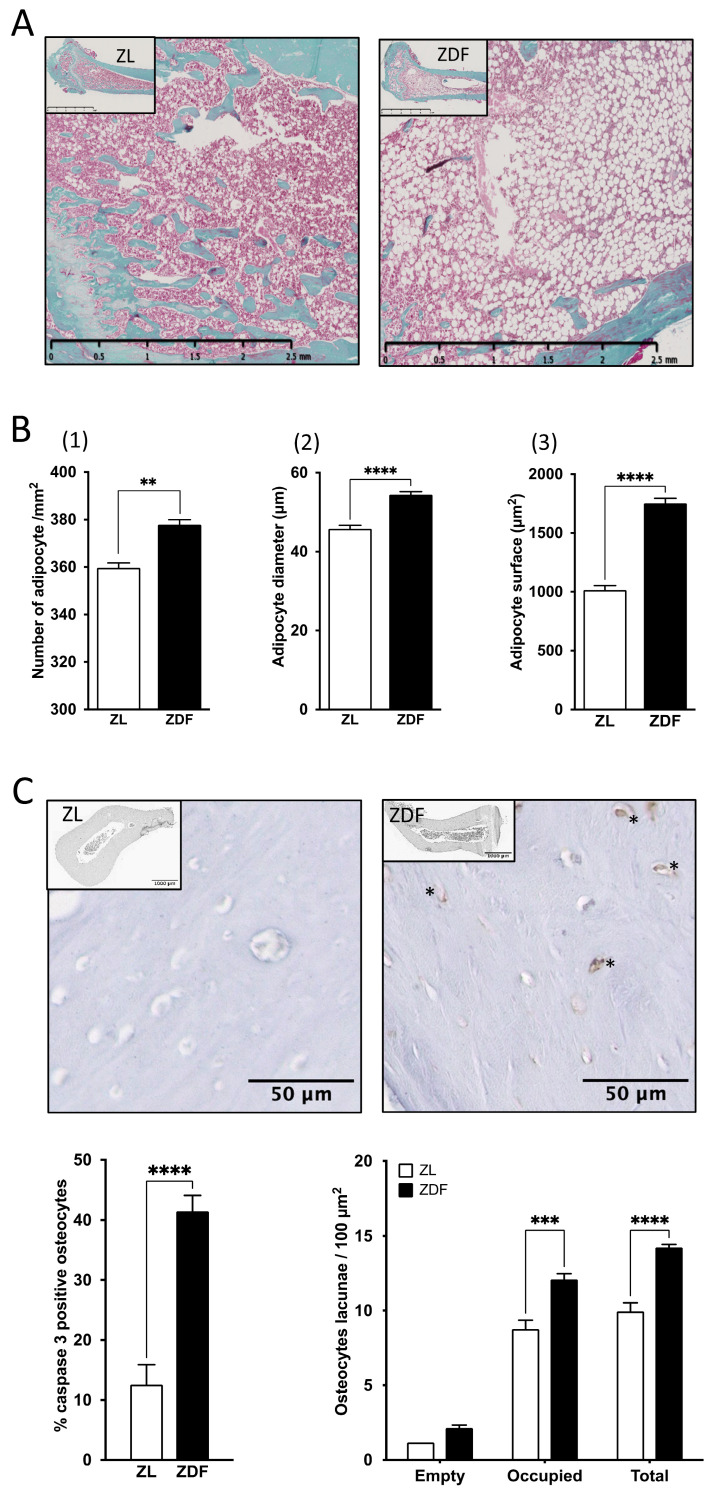
T2DM affects the bone marrow. (**A**) Representative images of decalcified histological sections of humeri from 24-week-old ZL and ZDF rats. Masson trichrome staining and standard error of the mean (SEM). The data are from 3 independent rats per condition (ZDF and ZL). ImageJ analysis scale bar = 2.5 mm. (**B**) (1) Number, (2) diameter, and (3) surface of adipocyte cells present in the bone marrow, analyzed with ImageJ on histological sections of humeri from ZDF and ZL rats (n = 3). (**C**) Representative images and analysis of immunohistochemical staining for caspase 3 in the humeri from ZDF rats. Analysis of caspase 3-positive osteocytes (star *) and osteocyte lacunae occupation in cortical bone from the humeri of ZL and ZDF rats. Values are expressed as the mean ± SEM from 3 to 5 independent regions of interest from 3 histological sections per rat (n < 9). * *p* < 0.05; ** *p* < 0.01; *** *p* < 0.001; **** *p* < 0.0001.

To assess changes in long-standing T2DM in the BMMSCs environment, we evaluated bone marrow cellularity parameters in ZDF rats. In the bone marrow of the ZDF rats’ humeri, the number of adipocytes per mm^2^ of bone marrow tissue area was higher (by 5%; *p* < 0.005), and the adipocyte diameter and surface were increased (by 18.8% and 72.6%, respectively; *p* <0.0001) compared with ZL rats (Figure 1A,B).

Moreover, the number of mononuclear cells harvested from the bone marrow of diabetic animals was significantly reduced (by 30%, *p* < 0.05, as previously observed [20]). In the ZDF group, the number of caspase 3-positive osteocytes significantly increased (by 229%; *p* < 0.0001) (Figure 1C). In addition, the number of osteocyte lacunae, normalized to the cortical bone area in humeral cross-sections, was significantly higher (by 30.1%; *p* < 0.0001) than in ZL controls.

### 3.3. AGEs Alter Select Functions of BMMSCs In Vitro

AGE accumulation in bone tissue has been reported as a characteristic feature of the local microenvironment in T2DM. Therefore, the effect of AGEs on the functionality of the BMMSCs harvested from ZL rats was compared to that on cells harvested from ZDF rats with long-standing T2DM.

**Figure 2:**
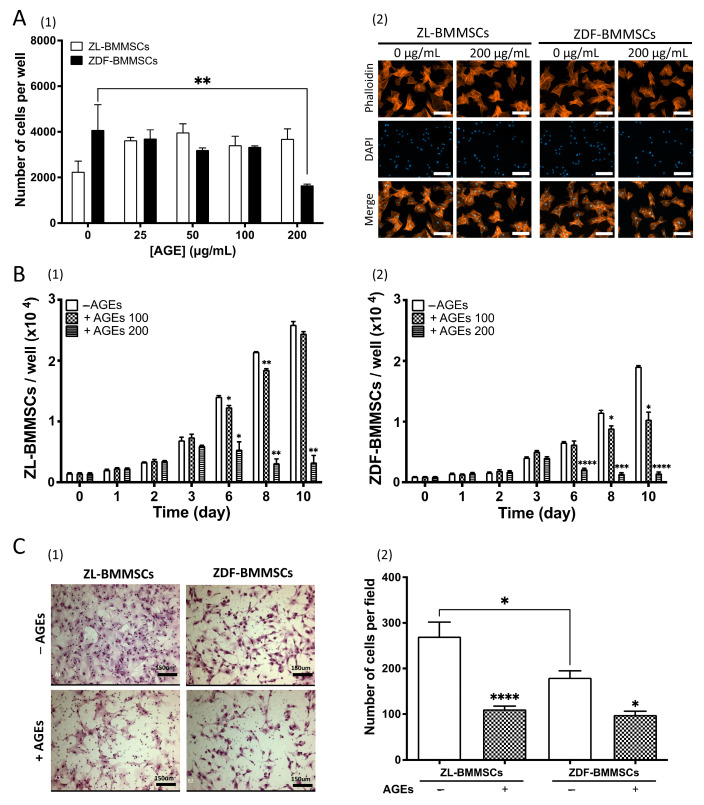
Impaired cell survival, proliferation, and migration of ZDF-BMMSCs treated with AGEs in normoglycemic conditions. (**A**) Cell survival of both ZL- and ZDF-BMMSCs in the absence or presence of up to 200 ug/mL AGEs-BSA added in serum-free aMEM was assessed by cell counting (1), and representative images with phalloidin staining (2) after 48 h exposure to AGEs-BSA. Scale bar: 200 mm. (**B**) Cell proliferation of (1) ZL- and (2) ZDF-BMMSCs cultured in the absence or presence of 100 or 200 ug/mL AGEs-BSA added to complete medium for up to 10 days. (1) Cell counting was performed with a DNA assay at the end points, using Cyquant^®^ fluorometric kit. (**C**) Migration potential of ZL- and ZDF-BMMSCs in the absence or presence of 100 µg/mL AGE-BSA in complete media. Images of migrated cells on the underside of the membrane with MGG staining (1). Cells were counted in five randomly selected, separate areas per membrane using ImageJ (2). Scale bar: 150 mm. Cell migration was determined using commercially available 24-well Boyden chambers. The data are from 3 wells per condition of 3 independent experiments (n = 9). Values are expressed as mean ± SEM. * *p* < 0.1; ** *p* < 0.01; *** *p* < 0.001; **** *p* < 0.0001.

To evaluate the effect of AGEs on BMMSCs survival, cells from ZL and ZDF rats were plated in a 2% FCS medium. Then, they were cultured under normoglycemic conditions in a serum-free medium, with or without AGEs (up to 200 μg/mL), for 2 days (Figure 2A). Under these conditions, AGEs did not affect the number of ZL-BMMSCs but had a cytotoxic effect on ZDF-BMMSCs, with a significant (*p* < 0.01) decrease in cell number (by 55%). Next, to assess the effect of AGEs on proliferation, BMMSCs were plated in supplemented with 2% FCS. Then, they were cultured under normoglycemic conditions in complete medium to ensure cell cycling and optimal expansion conditions with increasing concentrations of AGEs for up to 10 days. Under these conditions, AGEs significantly reduced (*p* < 0.001) the number of BMMSCs harvested from both ZL and ZDF rats (Figure 2B). The effect of AGEs on BMMSCs proliferation was dose-dependent and more pronounced in cells harvested from ZDF rats in comparison to those from ZL rats (Figure 2(B2)). At 100 µg/mL, the number of ZL-BMMSCs was slightly reduced, whereas the number of ZDF-BMMSCs was reduced by 45.7% (*p* < 0.1) at day 10. At 200 μg/mL, proliferation was markedly suppressed in both groups, with cell numbers reduced by 87.3% (*p* < 0.01) for ZL-BMMSCs and 92% (*p* < 0.0001) for ZDF-BMMSCs at day 10 (Figure 2B).

The migration potential of both ZL-BMMSCs and ZDF-BMMSCs was assessed using a Boyden chamber assay, with AGEs at 100 μg/mL in the bottom compartment. At the end of the experiment, the migrated cells were stained and analyzed using digital microscopy. Migrated BMMSCs from both ZL and ZDF rats were evenly distributed on the membrane surface. Migrated ZL-BMMSCs predominantly exhibited polygonal morphology with expanded cytoplasm, regardless of the presence of AGEs. In contrast, migrated ZDF-BMMSCs displayed greater morphological heterogeneity, including expanded, starlike, or even rounded forms (Figure 2(C1)). In the absence of AGEs, the number of migrated ZDF-BMMSCs was reduced (by 33.42%; *p* < 0.1) compared to that of ZL-BMMSCs. In the presence of AGEs, the number of migrated ZL-BMMSCs decreased by 59.03% (*p* < 0.0001) and the number of migrated ZDF-BMMSCs by 45.48% (*p* < 0.1) (Figure 2(C2)). AGES at concentrations up to 200 μg/mL further reduced the migration of ZL-BMMSCs.

To summarize, the findings show that AGEs exert a negative effect on select functions of BMMSCs cultured in vitro under normoglycemic conditions, and this effect is more pronounced on cells harvested from a diabetic microenvironment.

### 3.4. AGEs Modulate Differently Selected Gene Expression

BMMSCs harvested from the T2DM microenvironment have been shown to have an altered gene expression profile in addition to altered functionality [20]. Therefore, we investigated the effect of the AGEs-BSA on ZL-BMMSCs and compared it with the effect on ZDF-BMMSCs. To specifically address the key features of BMMSCs dysfunction in the context of T2DM, we selected a targeted panel of genes including IL1b, Cxcl1 (inflammation), Pparg, Runx2, ALP (lineage commitment), and RankL (bone remodeling). To this end, cells were cultured in the absence or presence of AGEs (100 μg/mL) added to a complete culture medium for 12 h and 48 h. This concentration of AGEs resulted in functional impairment without cytotoxicity (Figure 2B).

**Figure 3:**
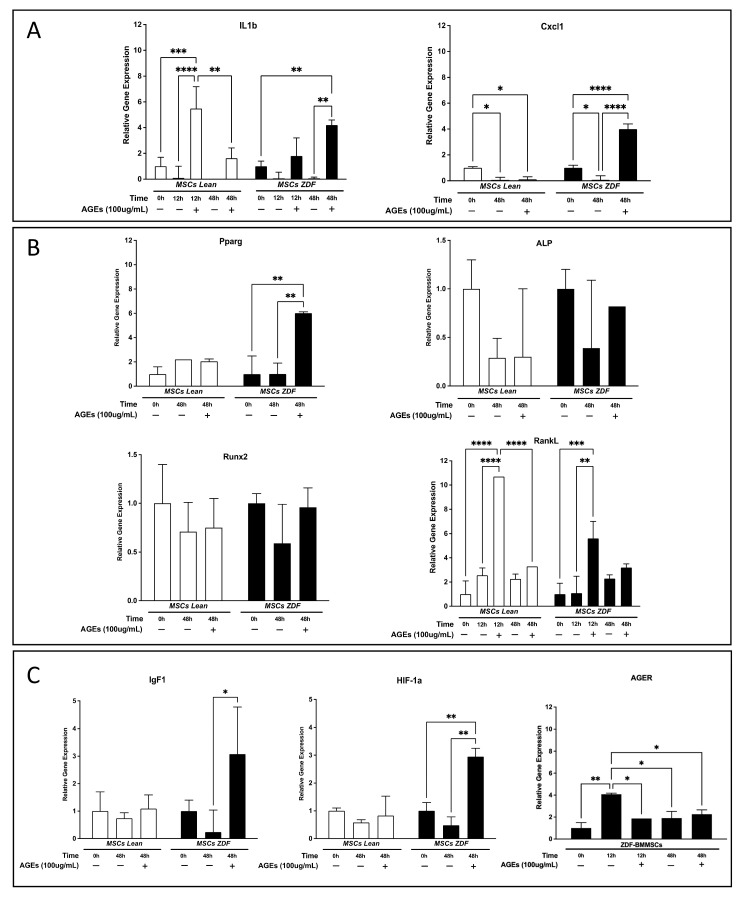
Expression of (**A**) select inflammation-related genes IL1b and Cxcl1; (**B**) adipo-osteogenic-related genes Pparg, ALP, Runx2, and RankL; (**C**) diabetic microenvironment-related genes IGF-1, HIF-1a and AGER from ZL- (white) and ZDF-BMMSCs (black) cultured under normoglycemic conditions in the absence or presence of 100 ug/mL of AGEs-BSA for 12 and 48 h. Values are expressed as the mean ± SEM of 3 independent wells (n = 3) from three independent experiments. The fold change in expression was analyzed based on the PCR cycle number (Ct). 18S rRNA was used as an endogenous control to normalize the data. Relative quantification of gene expression was performed according to the Pfaffl et al. method. Each reaction was performed in triplicate. * *p* < 0.05; ** *p* < 0.01; *** *p* < 0.001; **** *p* < 0.0001.

In ZL-BMMSCs, exposure to AGEs for 12 h resulted in a significant upregulation of IL1b (by 4.48-fold; *p* < 0.001), followed by a return to “basal” expression levels at 48 h (Figure 3A,B). The expression of the cxcl1 gene was downregulated in ZL-BMMSCs (by 0.9-fold; *p* < 0.05) at 48 h, regardless of the presence of AGEs (Figure 3A). In ZDF-BMMSCs, AGEs induced an upregulation of IL1b (by 3.2-fold; *p* < 0.01) at 48h and an upregulation of Cxcl1 (by 3-fold; *p* < 0.0001) at 12 h (Figure 3A).

In ZL-BMMSCs, exposure to AGEs for 12 h resulted in a significant upregulation of RankL (by 9.70-fold; *p* < 0001) gene expression, followed by a return to the “basal” expression level at 48 h (Figure 3A,B). In ZDF-BMMSCs, AGEs also induced an upregulation of RankL (by 4.6-fold; *p* < 0.001) at 12 h. Under these experimental conditions and in the absence of differentiation factors, AGEs modified the expression of Pparg, an adipogenic marker, only in ZDF-BMMSCs. After 48 h of exposure, AGEs led to a significant upregulation of Pparg (by 5-fold; *p* < 0.01) but did not alter the expression of select osteogenic markers, namely ALP and Runx2 (Figure 3B). In ZDF-BMMSCs, AGEs also upregulated the expression of diabetic microenvironment-related genes—IGF-1 by 11.83-fold (*p* < 0.05) and HIF-1a by 1.95-fold (*p* < 0.01)—after 48 h exposure (Figure 3C).

Moreover, ZL- and ZDF-BMMSCs showed differences in AGE-specific receptor (AGER) gene expression. At t0, the AGER gene was expressed only in ZDF-BMMSCs. In the absence of AGEs, the AGER gene expression increased at 12 h (by 3.08-fold; *p* < 0.01) and then decreased to the level observed at t0. At 12 h, in the presence of AGEs, AGER gene expression was similar to that observed at t0 (Figure 3C).

### 3.5. AGEs-Induced ROS Generation and Effects of N-Acetylcysteine (NAC)

It has been reported that AGEs increase ROS [15]. Therefore, ROS generation was evaluated in both ZL- and ZDF-BMMSCs, cultured in the presence (100 μg/mL) and absence of AGEs for 15 min. This concentration was selected based on dose–response experiments showing significant functional impairment without excessive cytotoxicity. For instance, a higher concentration (200 µg/mL) resulted in near-complete inhibition of proliferation (Figure 2B) and was therefore unsuitable for functional assays. DCFH-DA and DHE probes were used to evaluate intracellular ROS (iROS) and mitochondrial ROS (mROS), respectively.

**Figure 4:**
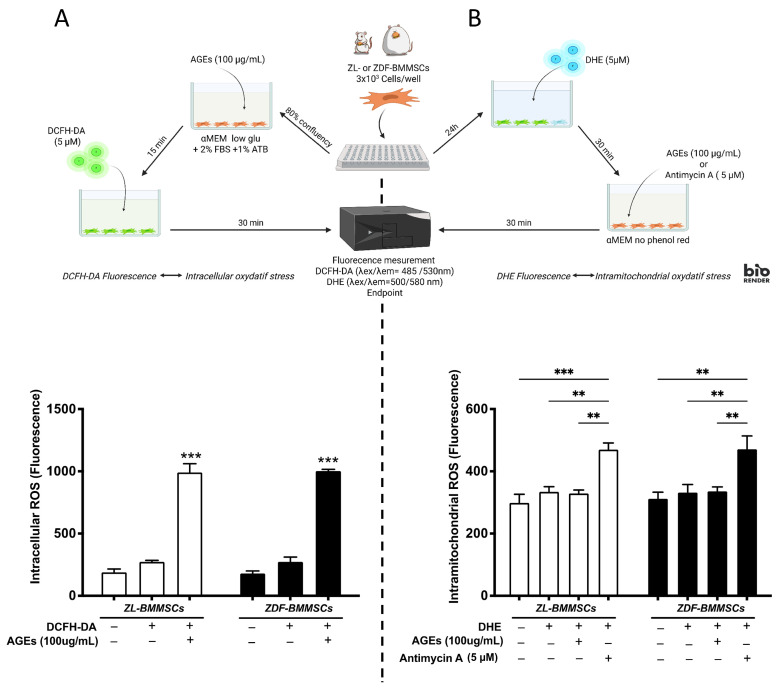
iROS in ZDF-BMMSCs and ZL-BMMSCs after exposure to 100 μg/mL of AGEs-BSA; values are expressed as mean ± SEM (n = 3). iROS measurements were performed at the endpoint using DCFH-DA (**A**) or DHE (**B**) fluorescent probes. Antimycin A was used as a positive control to block the respiratory chain for DHE measurements. Data are from 3 independent wells per condition and are representative of 2 independent experiments (n = 6). ** *p* < 0.01, *** *p* < 0.001. Created in BioRender. Landon, R. (2026) https://BioRender.com/wz09w95 (accessed on 31 March 2026).

The addition of AGEs into the medium resulted in a significant increase (by 270%; *p* < 0.001) in iROS (Figure 4A). In contrast, AGEs did not significantly affect mROS generation (Figure 4B), unlike Antimycin A (AMA), which was used as a positive control to block the respiratory chain in mitochondria. This suggests an intracytoplasmic localization of AGE-induced ROS. Furthermore, ZL- and ZDF-BMMSCs exhibited similar levels of AGE- and AMA-induced iROS and mROS.

To further characterize the AGE-induced iROS and the potential of an antioxidant to mitigate it, both ZL- and ZDF-BMMSCs were pre-treated or co-treated with N-acetylcysteine (NAC). T-buOOH and AAPH were used as control stressors to generate iROS. For the pre-treatment experiments, cells were incubated overnight with increasing concentrations of NAC before OS induction. For co-treatment experiments, 10 mM NAC was added simultaneously with the OS inducer.

**Figure 5:**
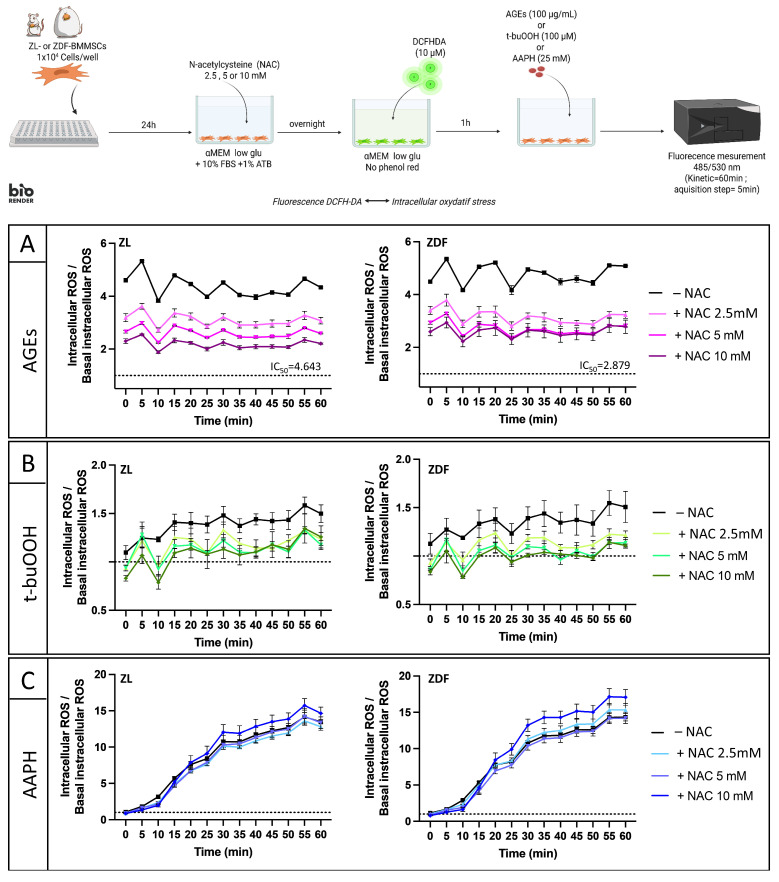
Impact of NAC pre-treatment on iROS levels in ZDF-BMMSCs and ZL-BMMSCs when exposed to (**A**) 100 μg/mL of AGEs-BSA, (**B**) 100 μM di-tert-butyl-peroxide (t-buOOH), and (**C**) 25 mM AAPH. Values are expressed as mean ± SEM (n = 3). iROS measurement was performed in a kinetic manner for 60 min using the DCFH-DA fluorescent probe. The data are from 3 independent wells per condition, representative of 2 independent experiments (n = 6). Created in BioRender. Landon, R. (2026) https://BioRender.com/wz09w95 (accessed on 31 March 2026).

In both ZL-BMMSCs and ZDF-BMMSCs, AGE-induced iROS increased from the beginning of the assay (>4 times higher than basal stress levels) and remained high for 1 h (Figure 5A). NAC pre-treatment significantly (*p* < 0.0001) reduced iROS in a dose-dependent manner in both cell populations, with ROS scavenging exceeding 40% at 10 mM NAC. However, lower concentrations of NAC were required for ZDF-BMMSCs compared to ZL-BMMSCs (IC50 2.9 vs. IC50 4.6). iROS levels induced by t-buOOH were lower than those induced by AGEs. They increased slightly to reach a plateau after 15 min in both cell populations (approximately 1.5 times the basal stress levels) (Figure 5B). In cells pre-treated with NAC, iROS production was reduced and more pronounced in diabetic cells. The generation of -iROS by AAPH was close to the basal level at t = 0 and increased linearly over time. After 60 min of incubation, a strong signal, nearly 15-fold higher than the basal stress state, was observed, indicating a higher iROS level than that observed with t-buOOH or AGEs. Pre-treatment with NAC did not protect the cells; instead, iROS levels remained high in both cell types (Figure 5C).

**Figure 6:**
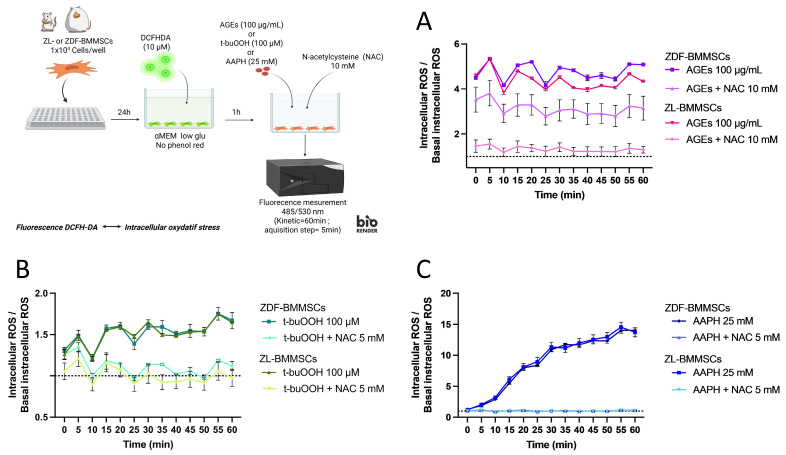
Impact of NAC co-treatment on ROS levels in ZDF-BMMSCs and ZL-BMMSCs when exposed to (**A**) 100 μg/mL of AGEs-BSA, (**B**) 100 μM t-buOOH, and (**C**) 25 mM AAPH. Values are expressed as mean ± SEM. (n = 3). iROS measurement was performed in a kinetic manner for 60 min using the DCFH-DA fluorescent probe. The data are from 3 independent wells per condition, representative of 2 independent experiments (n = 6). Created in BioRender. Landon, R. (2026) https://BioRender.com/wz09w95 (accessed on 31 March 2026).

To evaluate the cellular protection against OS by antioxidant co-treatment, ZL- and ZDF-BMMSCs were simultaneously exposed to an iROS inducer (AGEs, t-buOOH, or AAPH) and the antioxidant NAC (10 mM) (Figure 6). The level of iROS induced by AGEs was significantly reduced by NAC in ZL- and ZDF-BMMSCs (by ~70% and ~28%, respectively; *p* < 0.001 and *p* < 0.05) (Figure 6A). Adding NAC at the same time as AGEs resulted in a less effective reduction in AGE-induced ROS (>2-fold) in ZDF-BMMSCs compared to ZL-BMMSCs.

Under the same experimental conditions, co-treatment with NAC protected both cell types from t-buOOH- and AAPH-induced stress by inhibiting (*p* < 0.0001) the generation of iROS and maintaining the cellular basal stress state (Figure 6B,C).

In summary, iROS generation by AGEs was instantaneous, lasted for at least 60 min, and was higher than that observed with t-buOOH but lower than that observed with AAPH. Moreover, while the iROS generation was similar in both ZL- and ZDF-BMMSCs, it was modulated differently in response to NAC. Indeed, pre-treatment with NAC reduced iROS production in both ZL- and ZDF-BMMSCs in a dose-dependent manner, but the co-treatment showed a greater antioxidant potential in ZL-BMMSCs than in ZDF-BMMSCs.

## 4. Discussion

AGEs accumulate in the bone microenvironment and are considered central mediators of T2DM complications [14,28]. However, findings regarding their effects on BMMSCs are scarce and inconclusive. In the present study, AGEs were used to mimic a glycotoxic environment in vitro, and their effects on non-diabetic BMMSCs were compared with the effects on cells derived from long-standing T2DM bone marrow. Our results show that AGEs induce ROS generation and impair BMMSCs functions regardless of the context from which they were harvested. Notably, AGEs exerted a stronger effect and altered antioxidant defenses in cells harvested from a long-standing T2DM microenvironment, showing that prolonged exposure to hyperglycemic conditions impacts BMMSCs’ function and responsiveness to AGEs. Importantly, NAC reduced AGE-induced iROS, supporting its potential to mitigate OS in BMMSCs in the diabetic milieu.

In the present study, we found that long-standing T2DM was associated with significant alterations in the cellular composition of the bone marrow and surrounding matrix-embedded osteocytes, distinct from age-related changes. These changes may be associated with alterations in the trabecular and cortical bone microarchitecture previously described in the ZDF-T2DM model [23,29]. Compared with age-matched controls, the number of BMMSCs decreased in diabetic rats, and bone marrow adiposity increased, which probably resulted from adipocyte hypertrophy and hyperplasia. This finding is consistent with previous reports in TALLYHO/JngJ obese mice [30] and T2DM patients [31] showing that the T2DM microenvironment induces a shift in BMMSCs differentiation phenotype, favoring adipogenesis over osteogenesis [32]. In the T2DM-ZDF model, previous studies from our group have shown that BMMSCs cultured in vitro exhibit an increased propensity to differentiate into adipocytes, which is associated with upregulation of PPARγ and Adiponectin [20]. Similar results have been obtained in other T2DM rodent models and human T2DM-BMMSCs [33]. Moreover, an interesting finding in the present study is that, in the bone marrow surrounding matrix of the diabetic group, the density of osteocyte lacunae was higher, and the number of apoptotic osteocytes increased dramatically by 229% (*p* < 0.0001). These findings are consistent with reports of increased lacunar density [34] and sclerostin expression in the tibia of streptozotocin (STZ)-induced T1DM rats [35]. Likewise, an accumulation of senescent osteocytes has been observed in high-fat diet (HFD)/streptozotocin (STZ)-induced T2DM mice [36], and it has been associated with increased levels of CML, a component of AGEs, in both blood and bone tissue samples. Our findings corroborate literature evidence associating the expansion of bone marrow adiposity, altered BMMSCs fate, and osteocyte dysfunction in T2DM. Nevertheless, further investigation is required to ascertain the role of prolonged exposure to a hyperglycemic and AGE-rich environment in the observed alterations.

In the present study, AGE concentrations similar to those observed in the serum of diabetic patients [37], and in previous studies on stem cells [38], impaired differentially selected functions in both non-diabetic and diabetic BMMSCs. In line with reports in rat [39,40,41] and human BMMSCs [42] and adipose stem cells [43] harvested from a “non-diabetic” microenvironment, AGEs reduced ZL-BMMSCs proliferation and migration. AGEs also altered the expression profile of IL1b and Cxcl1 genes. Consistent with prior reports showing the regulation of inflammatory cytokines, including IL1b and Cxcl1, by AGEs via NF-κB in other cell populations, such as macrophages. Similar changes in select chemokine/cytokine expression have also been reported in human BMMSCs [41]. In ZL-BMMSCs, AGEs upregulated RankL expression, which may link AGE-induced inflammation to bone remodeling and suggest a possible contribution of AGE-exposed BMMSCs to a pro-resorptive microenvironment. Taken together, these findings suggest that AGEs could contribute to the reduced number of progenitor cells, as previously observed in the ZDF model [20].

More importantly, our results show, for the first time, that BMMSCs harvested from a T2DM microenvironment are more “sensitive” to AGE exposure, underscoring the vicious negative effect of the T2DM microenvironment. At first, under serum-deprivation conditions, while AGEs did not affect ZL-BMMSCs, they decreased ZDF-BMMSCs viability, which may indicate a differential response to serum components that interfere with AGE-mediated cell death. AGEs also more strongly impaired proliferation and migration in ZDF-BMMSCs than in aged-matched ZL controls. Moreover, the AGE-induced gene expression profile also differed between the two cell populations. For instance, AGE-induced IL1b and Cxcl1 expression exhibited distinct rates and kinetics in ZDF-BMMSCs in comparison to ZL controls. This probably translates to an altered inflammatory responsiveness in diabetic cells, possibly involving a delayed and amplified inflammatory profile. Furthermore, AGEs upregulated the Pparg gene only in ZDF-BMMSCs, indicating a diabetes-associated predisposition towards adipogenesis. In contrast, the expression of ALP and Runx2, markers of osteogenic commitment, remained unchanged, suggesting that AGEs promote lineage imbalance rather than differentiation inhibition. Nonetheless, to fully characterize how the AGE-rich, oxidative microenvironment modulates lineage commitment in BMMSCs, direct assessment under adipogenic and osteogenic induction conditions is required. Given that ZDF-BMMSCs under normoglycemic conditions also show increased apoptosis and reduced viability, proliferation, and migration, a permanent AGE-induced dysfunction in BMMSCs cannot be excluded. Additional studies are needed to determine whether AGE effects are transient or persistent, and a transcriptomic approach would provide a better characterization of AGEs’ impact on the BMMSCs phenotype.

Moreover, AGEs increased IGF-1 and HIF-1α expression, suggesting enhanced metabolic and stress-adaptive responses, and upregulated RAGE specifically in ZDF-BMMSCs. The transient increase in RAGE expression at 12 h, followed by a reduction at 48 h, may reflect an adaptive response to the culture environment [44,45]. However, RAGE expression at the protein level as well as RAGE-mediated signaling were not assessed, which limits the mechanistic insight of the present study.

AGEs accumulate in the T2DM microenvironment, modify the structure and function of proteins and lipids [14,28,46,47], and induce ROS production in many types of cells [48,49], contributing to OS [50]. In the present study, we investigated AGE-induced ROS in both non-diabetic and diabetic BMMSCs and compared the effects to those of stressors known to induce mitochondrial ROS (mROS) or cytoplasmic ROS (iROS). Our results show that AGEs primarily induced cytoplasmic ROS production, as no increase in mROS was observed. In non-diabetic BMMSCs, AGEs have been reported to increase iROS in parallel with increased apoptosis [39] and reduced proliferation [51,52]. Interestingly, similar levels of AGE-induced iROS were observed in both cell populations, indicating that the exacerbated deleterious effects of AGEs on diabetic BMMSCs are unlikely to be explained solely by iROS. It has been reported that in T2DM BMMSCs, NADPH oxidase is an important source of iROS implicated in redox signaling, and Nox4 expression is increased [53]. Further studies are needed to elucidate the signaling pathways underlying AGE-induced impaired functionality of BMMSCs.

In addition, this study is the first to demonstrate that NAC differently affects AGE-induced iROS in diabetic and non-diabetic BMMSCs under pre-treatment and co-treatment conditions. NAC is an antioxidant used to mitigate OS and its detrimental effects on MSCs sourced from various tissues [22,54,55]. It has been reported that NAC exerts antioxidant effects in non-diabetic BMMSCs under both normoxic [55] and hypoxic conditions [21]. In the present study, under pre-treatment conditions, NAC efficiently blocked AGE-induced iROS in both ZL- and ZDF-BMMSCs. The pronounced antioxidant effect of NAC observed can be attributed to its “indirect” effects. NAC enhances intracellular redox capacity prior to the sustained iROS production induced by AGEs. Indeed, NAC serves as a precursor to cysteine, promoting glutathione (GSH) synthesis and strengthening cells’ antioxidant defenses [55]. NAC can also activate the transcription factor Nrf2 [56], which regulates the expression of antioxidant and detoxification genes and inhibits the NF-κB [21] pathway, a central regulator of inflammation and stress responses. Under co-treatment conditions, NAC reduced iROS by 70% in non-diabetic BMMSCs, but by only 28% in diabetic cells. Because the direct scavenging of NAC is transient, its effectiveness may be insufficient under sustained ROS production, especially in ZDF-BMMSCs with reduced redox buffering capacity.

Moreover, NAC showed the strongest rescue effect against ROS generated by AGEs compared to t-BuOOH and AAPH under pre-treatment conditions. These differences likely reflect the distinct chemical nature, localization, and propagation of ROS, as well as the NAC’s rapid redox turnover, which limits its direct scavenging to transient effects. Its efficiency as a direct ROS scavenger depends on the kinetics and persistence of ROS production, while its indirect antioxidant effects require time and intracellular uptake. The absence of protection against AAPH-induced ROS under pre-treatment is consistent with the sustained generation of peroxyl radicals and extracellular lipid peroxidation that quickly exceeds cellular buffering capacity. Finally, the partial protection against t-BuOOH-induced radicals under pre-treatment aligns with the rapid propagation of t-BuOOH-driven oxidative chain reactions and hydroperoxide-dependent stress, which cannot be fully prevented by elevated basal glutathione alone. During co-treatment, NAC can also act as a transient radical scavenger, limiting early oxidative propagation and thus enhancing its protective effect.

The ability of NAC to protect cells cultured in vitro can be attributed to both its direct [56] and indirect [21,57] antioxidant properties. In the present study, functional rescue experiments—such as migration or proliferation following NAC treatment—were not performed, limiting our ability to show NAC-mediated benefit on diabetic BMMSCs’ functionality. Future studies incorporating such rescue assays will be essential to characterize the antioxidant protective potential of NAC in this context.

While this study provides valuable insights into the effects of long-standing T2DM on BMMSCs dysfunction and the role of AGE-induced OS, some limitations should be acknowledged. The preclinical design based on in vitro and ex vivo BMMSCs derived from a rat T2DM model may limit the translational interpretation of our findings. Osteogenic differentiation and in vivo bone regeneration were not directly assessed, which restricts the interpretation of the functional impact on bone repair. Finally, the molecular mechanisms underlying AGE-induced BMMSCs dysfunction were not fully elucidated, and further studies are required to clarify the involvement of specific signaling pathways such as the AGEs–RAGE axis.

## 5. Conclusions

In conclusion, this study showed that AGEs impair BMMSCs’ functionality and that long-standing diabetes increases these cells’ susceptibility to AGE-induced damage. NAC was found to reduce OS in BMMSCs harvested from a T2DM microenvironment. Overall, our study offers new insights into how the T2DM microenvironment alters BMMSCs function and highlights areas for further investigation of diabetes-associated complications.

This work is part of the PhD thesis of Rebecca Landon, conducted at Université Paris Cité, 2023 [58].

## Figures and Tables

**Table 1:** Blood parameters in ZDF and ZL rats. Values are from 3 independent experiments, n = 9 rats/condition. ** *p* < 0.01; *** *p* < 0.001 and **** *p* < 0.0001.

	ZL		ZDF		ZDF vs ZL
	Mean	SEM	Mean	SEM	*p* Value
Glycated hemoglobin (%)	2.90	0.23	11.90	2.01	**
Alkaline Phosphatase (U/L)	77.00	3.87	339.50	47.10	***
Glucose (mmol/L)	16.55	1.39	40.68	1.30	****
Cholesterol (mmol/L)	2.21	0.06	5.43	0.23	****
Triglycerides (mmol/L)	1.24	0.11	3.05	0.23	****
Fructosamin (mmol/L)	0.23	0.01	0.33	0.01	****
Ultra HDL (mmol/L)	0.63	0.02	1.73	0.03	****
LDL-C (mmol/L)	1.00	0.06	2.33	0.19	****
Phosphorus (mmol/L)	1.91	0.10	2.04	0.18	ns
Calcium (mmol/L)	2.55	0.04	2.61	0.08	ns
Sodium (mmol/L)	13811	1.12	134.22	1.64	ns
Potassium (mmol/L)	4.97	0.38	4.67	0.55	ns

## Data Availability

The original contributions presented in this study are included in the article. Further inquiries can be directed to the corresponding authors.

## Abbreviations

The following abbreviations are used in this manuscript:

AGEs	Advanced Glycation End Products
AGEs-BSA	Advanced Glycation End Products–Bovine Serum Albumin
Ager	Gene Coding for Advanced Glycation End Product Receptor
ALP	Alkaline Phosphatase
AMA	Antimycin A
AAPH	2,2′-Azobis(2-amidinopropane) dihydrochloride
ATB	Antibiotics/Penicillin–Streptomycin
BM	Bone Marrow
BMMSCs	Bone Marrow Mesenchymal Stromal Cells
CFU-F	Colony-Forming Unit–Fibroblast
CO_2_	Carbon Dioxide
Cxcl1	Chemokine (C-X-C motif) Ligand 1
DAB	3,3′-Diaminobenzidine
DCFH-DA	Dichloro-dihydro-fluorescein diacetate
DHE	Dihydroethidium
EDTA	Ethylenediaminetetraacetic acid
FCS	Fetal Calf Serum
HbA1c	Glycated Hemoglobin
HFD	High-Fat Diet
HIF-1α/Hif-1a	Hypoxia-Inducible Factor 1 alpha
HRP	Horseradish Peroxidase
IGF-1	Insulin-like Growth Factor 1
IL1β/IL1b	Interleukin 1 beta
iROS	Intracellular Reactive Oxygen Species
LDL-C	Low-Density Lipoprotein Cholesterol
mROS	Mitochondrial Reactive Oxygen Species
MSCs	Mesenchymal Stem Cells
NAC	N-acetylcysteine
NF-κB	Nuclear Factor kappa B
Nox4	NADPH Oxidase 4
OS	Oxidative Stress
PBS	Phosphate-Buffered Saline
Pparg/PPARγ	Peroxisome Proliferator-Activated Receptor gamma
RAGE	Receptor for Advanced Glycation End Products
RANKL	Receptor Activator of Nuclear Factor Kappa-B Ligand
ROI	Region of Interest
ROS	Reactive Oxygen Species
RT-PCR	Reverse Transcription Polymerase Chain Reaction
Runx2	Runt-related Transcription Factor 2
SEM	Standard Error of the Mean
STZ	Streptozotocin
T2DM	Type 2 Diabetes Mellitus
t-BuOOH	tert-Butyl Hydroperoxide
TBST	Tris-Buffered Saline with Tween
ZDF	Zucker Diabetic Fatty
ZL	Zucker Lean
